# Iron Deficiency and Iron Deficiency Anemia in Children With First Attack of Seizure and on Healthy Control Group: A Comparative Study

**Published:** 2014

**Authors:** Razieh FALLAH, Behnaz TIRANDAZI, Farzad FERDOSIAN, Nafiseh FADAVI

**Affiliations:** 1 Department of Pediatric Neurlogy, Growth Disorders of Children Research Center, Shahid Sadoughi University of Medical Sciences, Yazd, Iran; 2 Shahid Sadoughi University of Medical Sciences, Yazd, Iran; 3 Subspecialty in Pediatric Infectious Disease, Department of Pediatrics, Shahid Sadoughi University of Medical Sciences, Yazd, Iran

**Keywords:** Seizure, Febrile Seizure, First Unprovoked Seizure, Iron deficiency, Iron deficiency anemia

## Abstract

**Objective:**

Seizures are the most common pediatric neurologic problem. Research of the association between iron deficiency and seizures has shown conflicting results.

This study evaluates iron status of children with a first seizure attack (febrile seizure (FS) or first unprovoked afebrile seizure (FUS) and healthy control group.

**Materials & Methods:**

In a cross sectional case control study, iron status of 6–60 month year old admitted children with first seizure to Shahid Sadoughi Hospital from August 2011–December 2012 were evaluated and compared with healthy control children that were referred to primary health care center of Azadshar, Yazd, Iran.

**Results:**

150 children were compared in three equal (FS, afebrile seizure, and control) groups.

Hemoglobin levels in FUS (11.39 ± 1.07 g/dl) and FS (11.46 ± 1.18 g/dl) were lower than the control group (11.9 ± 0.89 g/dl) group.

Serum iron levels in FS (38.52 ± 11.38 μg/dL) and FUS (42.68 ± 14.76 μg/dL) were lower than the control group (54.32 ± 13.46 μg/dL).

Serum ferritin level in FUS (46.21 ± 27.63 ng/mL) and FS (48.91 ± 22.96 ng/ mL) was lower than the control group (75.13 ± 35.57 ng/mL).

Iron deficiency (48% in FS, 44% in FUS and 28% in control group) and iron deficiency anemia (26% in FUS, 22% in FS, and 10% in healthy children) was more frequent in children with seizures.

**Conclusion:**

Iron status should be evaluated in children with a first attack of febrile or afebrile seizures.

## Introduction

Seizures are the most common pediatric neurology problem that occurs in 4–10% of children in the first 16 years of their life and febrile seizures (FS) are the most common type of childhood seizures that occur in 2–5% of neurologically healthy children (1).

Iron deficiency is the most common micronutrient deficiency that affects at least one third of the world’s population. Anemia is the most common clinical manifestation of iron deficiency, but other organs and systems may also be affected. 

Cognitive dysfunction, psychomotor retardation, behavioral problems, pica, breath holding spells, restless leg syndrome, and thrombosis could be associated with iron deficiency. The effect of iron deficiency in a developing brain and mechanisms such as altered development of hippocampus neurons, impairment of energy metabolism, delayed maturation of myelin, slowed visual and auditory evoked potentials, and alterations in synaptic neurotransmitter systems including norepinephrine, dopamine, glutamate, gamma-aminobutyric acid (GABA), and serotonin may be responsible for these symptoms (2,3).

Research regarding association of iron deficiency and seizures has showed conflicting results with most comparing the iron status in children with febrile seizure and febrile children without seizure.

This study compares the iron status of children with the first attack of seizure [febrile seizure (FS) or first unprovoked afebrile seizure (FUS)] and a healthy control group in Yazd, a city in central of Iran.

## Material & Methods

This is a cross sectional case control study by sample size based on Z formula and confidence interval of 95% with 80% power, type one error of 5% to detect any significant differences between the groups with a level of 0.05 for which 50 children per group (febrile seizure, first unprovoked seizure, and healthy children) were assessed and iron status was evaluated. 

The seizure group consisted of 100 aged 6–60 months admitted children with first attack of seizure (FS or FUS) to Shahid Sadoughi Hospital Yazd, Iran from August 2011–December 2012.

FS has been defined as a seizure associated with a febrile illness in the absence of central nervous system infection or acute electrolyte abnormalities in 6–60 month-old children without previous afebrile seizures. FS is further classified as simple and complex. Complex FS is defined as a seizure lasting more than 15 minutes and recurring within 24 hours or focal seizure (1). 

The control group consisted of healthy age and sex matched control children that were referred for routine health care to a primary health care center in Azadshar. 

The exclusion criteria consisted of receiving an iron combination within the past month, the presence of any chronic systemic diseases (cardiac, renal, metabolic, malignancy, and rheumatologic, among others) and having neurodevelopmental delay, previous afebrile seizure, or acute central nervous system infection (meningitis, encephalitis).

A venous blood sample was obtained from all the children in all groups and hemoglobin (Hb) level, Hematocrit (Hct), mean corpuscular volume (MCV), serum ferritin level, serum iron level, and total iron-binding capacity (TIBC) were measured for all children in the Shahid Sadoughi Hospital laboratory. Anemia was defined as Hb level of less than 10.5g/dl in 6 months to 2 years and less than 11.5g/dl in 2 to 6 years. 

Iron deficiency was defined as serum ferritin level of less than 12 ng/mL if CRP was negative or 1+, ferritin level of less than 30 ng/mL if CRP was ≥ 2+, or serum iron levels of less than 22μ g/dL or transferrin saturation (a percentage calculated as serum iron concentration/TIBC × 100) of less than 16%. The criterion had a sensitivity of 75% and a specificity of 76% (5-8).

The data were analyzed using SPSS 17 statistical software. A Chi-square test was used for data analysis of qualitative variables and mean values were compared using an ANOVA test. Differences were considered significant at P-values of less than 0.05. 

Informed consent was taken from the parents and the study was approved by the Ethics Committee of Shahid Sadoughi University of Medical Sciences, Yazd, Iran. 

## Results

150 children including 71 girls and 79 boys with mean age of 2.04 ± 1.2 months were compared in three equal (FS, FUS, and control) groups.

In the FS group, 18 (36%) children had complex FS. 

Among those with complex FS, three children had multiple seizures within 24 hours, 13 had focal features, and 2 had prolonged convulsions. Table 1 shows a comparison of demographic characteristics of the children in the three groups, which indicates no statistical significant difference evident from gender distribution, positive family history of epilepsy, and mean age and weight. However, a positive family history of FS was more frequent in children with FS. Table 2 shows a comparison of laboratory characteristics of children in the three groups that indicate Hb level and serum ferritin level in children with afebrile and FS was lower than for the healthy group and serum iron levels in children with febrile and afebrile seizure were lower than in the control group. Nevertheless, no statistical significant differences were seen from hematocrit, MCV, and TIBC.

Table 3 shows a comparison of frequency of iron deficiency and iron deficiency anemia in the three groups, which indicates that iron deficiency and iron deficiency anemia are more frequent in children with a first seizure.

Table 4 shows comparisons of type and duration of seizures in children with and without iron deficiencies and indicates that in children with iron deficiency seizures were longer.

## Discussion

In this study, the iron status of children with first attack of febrile or afebrile seizure and healthy control children were compared. 

Children with first seizure, hemoglobin levels, serum iron levels, and serum ferritin levels were lower than in the control group and iron deficiency (48% in FS, 44% in afebrile seizure, and 28% in control group) and iron deficiency anemia (26% in afebrile seizure, 22% in FS, and 10% in healthy children) were more frequent in children with seizures.

Modaresi et al indicated that in Isfahan, Iran, the mean Hb, HCT, ferritin, iron, and MCH were lower in FS children than in febrile children without seizure (9). 

Zareifar et al indicated that in Shiraz, Iran, iron deficiency (56.6% vs. 24.8%) was more frequent in children with FS and in the FS group; Hb levels were lower than in febrile children without seizure (10).

Akbayram et al indicated that in Turkey, in children with FS, serum iron levels were lower than in the healthy children group (11).

Kumari et al indicated that in India, in FS children, iron deficiency was more frequent than in febrile children without seizure (6).

In another Indian study, the mean of serum ferritin levels were significantly lower in FS children than in children with febrile illness but without convulsion (12). 

Sherjil et al indicated that in Pakistan, in children with FS, iron deficiency anemia was more frequent than in febrile children without seizures (31.58% vs. 19.6%) (13).

In a study conducted in Islamabad, children with FS had hemoglobin, hematocrit, and serum ferritin level that were significantly lower than in febrile children without seizures (14).

In a Canadian study, iron deficiency (9% vs. 5%) and iron deficiency anemia (6% vs. 4%) were more frequent in children with FS and the odds ratio for iron deficiency in patients with FS was 1.84 (15).

However, Derakhshanfar et al study indicated that in Mofid hospital, Tehran, Iran, FS children Hb, Hct, MCV, serum iron, and plasma ferritin were higher and TIBC was lower than in febrile children without seizures and iron deficiency anemia was more frequent in the control group (16).

Bidabadi indicated that in Rasht, Iran FS children serum iron and plasma ferritin were significantly higher and TIBC was significantly lower than in febrile children without convulsions (17).

Abaskhanian indicated that in Sari, Iran, FS children had serum iron levels that were significantly higher, and iron deficiency anemia was less frequent (42% vs. 60%) than in febrile children without seizures (18). Kobrinsky indicated that in Fargo, in children with FS, iron deficiency was less frequent, but Hb, hematocrit, and MCV were higher and suggested that iron deficiency anemia might protect children against FS (19).

**Table 1:** Comparison of Demographic Characteristics of Children in Three Groups

**Group**		**Febrile seizure**	**Afebrile seizure**	**Healthy control group**	**P.value**
**Data**					
**Sex**	**Girl**	21	26	24	0.61
	**Boy**	29	24	26	
**Family history of febrile seizure in first and second degree family **	**Yes**	15	4	8	0.03
	**No**	35	46	42	
**Family history of epilepsy in first and second degree family**	**Yes**	4	3	2	0.71
	**No**	46	47	48	
**Age in month (mean ±SD)**		22.78± 11.08	23.18± 12.94	24.64± 12.28	0.52
**Weight in kilogram (mean ±SD)**		11.66 ± 3.21	11.94 ± 4.38	12.61± 4.03	0.26

**Table 2:** Comparison of Laboratory Characteristics of Children in Three Groups

**Group**	**Febrile seizure ** **Mean ± SD**	**Afebrile seizure** **Mean ± SD**	**Control group ** **Mean ± SD**	**P.value**
**Data**				
**Hemoglobin level in g/dl (mean ±SD)**	11.46 ± 1.18	11.39 ± 1.07	11.9 ± 0.89	0.039
** Hematocrit in percent (mean ±SD)**	34.22 ± 3.23	33.83 ± 3.11	35.08 ± 2.83	0.118
** MCV in μm3 or fL (mean ±SD)**	75.51 ± 5.11	76.03 ± 7.88	75.12 ± 7.37	0.815
**Serum ferritin level in ng/mL (mean ±SD)**	48.91 ± 22.96	46.21 ± 27.63	75.13 ± 35.57	0.001
**Serum iron levels in μg/dL(mean ±SD)**	38.52 ± 11.38	42.68 ± 14.76	54.32 ± 13.46	0.003
**TIBC in μg/dL**	343.88 ± 72.25	337.48 ± 68.96	341.62 ± 51.51	0.882

**Table 3:** Comparison of Frequency of Iron Deficiency and Iron Deficiency Anemia in Three Groups

**Group**		**Febrile seizure**	**Afebrile seizure**	**Control group**	**P.value**
**Data**					
**Iron deficiency **	**Yes**	24	22	14	0.03
	**No**	26	28	36	
**Iron deficiency anemia**	**Yes**	11	13	5	0.04
	**No**	39	37	45	

**Table 4:** Comparison of Type and Duration of Seizure in Children With and Without Iron Deficiency

**Group**		**With iron deficiency**	**Without iron deficiency**	**P.value**
**Data**				
Type of seizure	Generalized	31 (43.7%)	40 (56.4%)	0.46
	Partial	15 (51.7%)	14 (48.3%)	
Duration of seizure in minute (mean ±SD)		7.52 ± 3.77	5.17 ± 2.95	0.01

Talebian et al indicated that in Kashan, Iran, the risk of FS occurrence in anemic children seemed to be less than that in children without anemia (20).

The frequency of iron deficiency anemia in FS children and febrile children without seizure was not significantly different in Momen (21), in Salehi (22), and Amirsalari (23) studies.

Iron status in afebrile seizure was evaluated in two other Iranian studies (24, 25).

In a study in Kerman, Iran, serum iron and TIBC of 11-41 year old patients with first attack of generalized tonicclonic seizures were higher than in the control group (24).

Asadi-Pooya indicated that the means of Hb levels, hematocrit, and MCV were not statistically different in epileptic patients and the control group (25). 

In a study in Kenya (a malaria endemic area), the frequency of iron deficiency was not different in 3–156 month old children with acute seizures and the control group (33.8% vs. 27.1%). However, in their metaanalysis of 8 case-control studies, iron deficiency was more frequent in children with acute FS than in the control (30.5% vs. 21.9%) group (4). 

Possible explanations for these discrepancies are differences in: age, nutritional habits, geographic area, sample sizes, and the control group. 

Iron deficiency and iron deficiency anemia may play an important role in inducing seizures from the following mechanisms:

1. Decrease of GABA inhibitory neurotransmitter due to change in its metabolism;

2. Change in neuron metabolism; 

3. Reduction of enzymes such as monoamine and aldehyde oxidases; and,

4. Impairment in oxygenation and energy metabolism of the brain (26).

In conclusion, iron deficiency and iron deficiency anemia were more frequent in children with first attack of febrile or afebrile seizure, iron deficiency may be an important risk factor for the development of seizures, and the evaluation of iron status could be done in children with first attack seizures.
